# Effects of Data Aggregation on Time Series Analysis of Seasonal Infections

**DOI:** 10.3390/ijerph17165887

**Published:** 2020-08-13

**Authors:** Tania M. Alarcon Falconi, Bertha Estrella, Fernando Sempértegui, Elena N. Naumova

**Affiliations:** 1 Division of Nutrition Epidemiology and Data Science, Friedman School of Nutrition Science and Policy, Tufts University, Boston, MA 02111, USA; elena.naumova@tufts.edu; 2 Department of Immunology, Faculty of Medical Sciences, Central University, Quito 170136, Ecuador; bmestrella@uce.edu.ec (B.E.); fersempert@biociencias-ceb.org (F.S.)

**Keywords:** data aggregation, time series, harmonic regression, seasonality, infectious diseases

## Abstract

Time series analysis in epidemiological studies is typically conducted on aggregated counts, although data tend to be collected at finer temporal resolutions. The decision to aggregate data is rarely discussed in epidemiological literature although it has been shown to impact model results. We present a critical thinking process for making decisions about data aggregation in time series analysis of seasonal infections. We systematically build a harmonic regression model to characterize peak timing and amplitude of three respiratory and enteric infections that have different seasonal patterns and incidence. We show that irregularities introduced when aggregating data must be controlled during modeling to prevent erroneous results. Aggregation irregularities had a minimal impact on the estimates of trend, amplitude, and peak timing for daily and weekly data regardless of the disease. However, estimates of peak timing of the more common infections changed by as much as 2.5 months when controlling for monthly data irregularities. Building a systematic model that controls for data irregularities is essential to accurately characterize temporal patterns of infections. With the urgent need to characterize temporal patterns of novel infections, such as COVID-19, this tutorial is timely and highly valuable for experts in many disciplines.

## 1. Introduction

Seasonal infections, like respiratory and enteric infections, have well-known seasonal patterns which may vary by the pathogen [1,2,3], population [4], and location [2,5]. Common infections, such as giardiasis, have a single annual peak in incidence [6,7], while others, like cryptosporidiosis, demonstrate two peaks in certain locations [7,8]. Epidemiologists study those temporal patterns using time series analysis techniques that are widely described in the literature [9,10,11,12,13,14,15,16]. However, models with higher levels of mathematical complexity remain largely underutilized, even if they offer advantages over simpler models for analyzing seasonal infections. Characterization of seasonality for legionellosis, for example, has been typically limited to a simple description of the month with high incidence. The highest number of cases were reported between June and October in the U.S. [17], July and August in Canada [18], and August and November in Europe [19]. This non-parametric approach uses an indicator variable that groups data into categories reflecting time periods (e.g., months) [10]. The use of coarse grouping is simple to apply and easy to interpret, but it prevents a fully detailed, accurate, and comprehensive analysis of seasonal patterns. The inclusion of finer seasonal categories could lead to over-parameterization of the model. Harmonic time series analysis offers a clear advantage over the non-parametric indicator variable approach because it uses only two parameters (sine and cosine terms) to characterize essential aspects of seasonality: a point in time when the seasonal curve reaches its maximum (i.e., peak timing), the amplitude of that peak, and the duration of the seasonal increase [9]. A study of influenza in Brazil, for example, used harmonic regressions to characterize seasonal amplitude and peak timing in each state [20]. Those seasonal characteristics revealed that influenza traveled as a seasonal southward wave across Brazil. Another study used harmonic regressions to characterize the seasonality of hospitalization records of Salmonella in the U.S. and found three potential geographic targets for disease interventions [21]. Characterizing essential aspects of seasonality, such as peak timing and amplitude, can lead to important insights on the epidemiology of infections.

In epidemiological studies, time series analysis is typically conducted on aggregated counts (e.g., weekly or monthly), although data tends to be collected at finer temporal resolutions (e.g., hourly or daily) [22,23,24]. Particularly when analyzing secondary data, such as data from surveillance systems, researchers may not have access to the finer resolution data. For example, publicly available incidence data from the Centers for Disease Control and Prevention (CDC) are usually only available as weekly or monthly disease counts [25]. When finer resolution data are available, researches often still choose to aggregate data into coarser time units. Aggregation is often used to reduce noise in the data, to reflect underlying biological processes, or to maintain confidentiality, but it should be done cautiously [26]. To aggregate data, a researcher must decide on the temporal reference that will be used to build the time series (e.g., calendar or study), the temporal unit that will be used for the analysis (e.g., months), and the definition of that unit (e.g., how many days in a month) [27]. A calendar time series uses calendar information as the corresponding temporal reference and often has predetermined definitions of the temporal unit (e.g., number of days in Gregorian calendar months). A study time series uses study characteristics, like start and end times, as a reference. The definition of a week (e.g., when does it start), of a month (how many days are in a month), and of a season (start date and duration) could significantly vary based on the temporal reference selected and study characteristics, such as geographic location. Furthermore, it is also important to consider during data aggregation additional local calendar characteristics, such as non-working days and holidays, which may impact data collection and analysis [28]. The effects of calendar characteristics during data aggregation are rarely discussed in epidemiological literature even though they have been shown to impact model results [29,30].

In this step-by-step tutorial, we aim to demystify and make harmonic regression more easily accessible to scientists and epidemiologists, while emphasizing the potential effects of data aggregation. Each modeling step includes a critical thinking process for making decisions about data aggregation that may impact model results, including the selection of a temporal reference and definition of temporal units. With the urgent need to characterize temporal patterns of novel infections, such as COVID-19, this tutorial is not only timely but also highly valuable. As an illustrative example, we use historical data from a multi-year study of the incidences of acute upper respiratory infections (AURI), acute lower respiratory infections (ALRI), and diarrhea in urban children in Quito, Ecuador. These diseases are representative of common seasonal respiratory and enteric infections that are widely studied in the literature. We provide a de-identified sample dataset and R code as part of this tutorial. We start by building the time series of disease counts based on daily, weekly, and monthly data aggregation. We explore data irregularities, including the effects of the day of the week. We observe differences in distributions between rare (ALRI) and common (AURI and diarrhea) diseases, and differences due to data aggregation. We then build a time series model accounting for trend and seasonal patterns, and we show how to characterize seasonal peak timing and amplitude. We provide a de-identified sample dataset (Data S1) and R code (Code S1) as part of this tutorial. Throughout this process, we check the stability of findings across years and offer recommendations for sensitivity analysis. Time series analysis is essential for processing both longitudinal cohort measurements and surveillance records, thus we aimed this tutorial for a broad range of analytical tasks.

## 2. Case Study Data

For this tutorial, we used data from a longitudinal cohort study conducted between 2000 and 2005 in Quito, Ecuador. The study consisted of four sequential cohorts, with new children aged 6 to 36 months being enrolled in July of each year. Children in each cohort were observed by nurses and physicians for diarrheal and respiratory (ALRI and AURI) infections four days per week for up to 50 weeks. Examples of AURI included what are known colloquially as colds, while ALRI included the less common but more medically serious infections such as pneumonia. Diarrhea was defined as three or more loose bowel movements over a 24-hour period. Out of all 2582 children enrolled over the course of the study, 2136 children (82.7%) completed the study. The study was registered at www.ClinicalTrials.gov NCT00228254 and was conducted in accordance with the Declaration of Helsinki. Informed parental consent was obtained for each child. The Ethical Committee of the Corporación Ecuatoriana de Biotecnología and the Tufts University School of Medicine Institutional Review Board approved and monitored the study. For this retrospective cohort analysis, the lead researcher provided access to a database that had a compilation of de-identified daily data relevant to this analysis. We used R version 3.6.2 [31] and RStudio Version 1.2.5033 [32] for all data processing and statistical analyses. We provide a de-identified sample dataset (Data S1) and R code (Code S1) as part of this tutorial.

## 3. Building the Time Series

A time series is a sequence of values of a random variable that are ordered with respect to a temporal reference. In epidemiology, a time series often reflects temporal variations in disease prevalence or incidence. To create a time series of infection counts, individual occurrences within a specific time period must be compiled into *y_t_*, which represents the number of events (i.e., event counts) observed during time unit *t*. The point designated as the beginning of time, *t* = 1, and the definition of units of time (e.g., months) depend on the study design and temporal reference. A calendar time series uses calendar information as the corresponding temporal reference. A study time series uses study characteristics, such as start and end times, as a reference. The selection of a temporal reference has important implications for time series analysis. The effects of different temporal references are visible when aggregating counts over time. Table 1 shows the organization of a time series for diarrheal counts and contains time elements needed for future analysis: the calendar date, day of the week, and days with respect to the calendar and study time series. Specifically, counts of infections were first monitored on Thursday, 20 July 2000, the first day of the study time series. If using a calendar as a reference point, the first day of the week, Sunday, 16 July, will be considered the first day of the time series. Note that in other locations, the first day of the week could be a Monday and not a Sunday. If counts are aggregated into weeks (seven days in a week), week 1 will have 46 diarrheal cases for the study time series and 25 cases for the calendar time series (Table 1). In the latter case, week 1 would be considered incomplete. The discrepancy of those two numbers results from the first four days of week 1 of the calendar time series having no data. That first calendar week would, therefore, be considered incomplete. In this tutorial, we will use a study time series (henceforward referred to simply as time series) and not a calendar time series to avoid introducing additional irregularities (e.g., incomplete weeks) into our data. Regardless of the temporal reference used to build the time series, we must consider data irregularities during the design, collection, and analysis of time-series data. We further discuss approaches to characterize and model data irregularities in Section 4.1, Section 4.2 and Section 7.3.

Our case study was designed to collect data for 50 weeks starting in July of each year. The first day of data collection was considered as *t=1* for each cohort (for example, Thursday, 20 July 2000 for Cohort 1). We compiled the individual records of episodes of diarrhea, AURI, and ALRI as daily counts for each of the four cohorts and then aggregated data into weekly counts. Table 1 shows the organization of the time series for diarrheal counts, as an illustrative example, and contains time elements needed for future analysis: the calendar date, day of the week, and days with respect to the time series. To aggregate daily counts, we defined a week as seven consecutive days and a month as four consecutive weeks or 28 days, irrespective of the Gregorian calendar months (Table 1 and Table 2). The Gregorian calendar has different months with different numbers of days (28 or 29 days for February and 30 or 31 days for all other months), which might be non-trivial [30,33]. To avoid this problem, the aggregated monthly data can be divided by month length and multiplied by 30.4375 (365.25/12), the average month length [34]. Another solution, which is used in this tutorial, is to divide a year into thirteen 28-day periods instead of calendar months to follow a moon cycle.

When deciding to aggregate data, the effects of aggregation on sample size should be taken into consideration. For time series analysis, the sample size depends on the length of the study period, the selected temporal unit of analysis, and completeness of time-referenced records. Aggregation of data to a coarser temporal unit, for example from days to months, ultimately reduces the sample size and could result in reduced statistical power. The sample size, *N*, in our case study goes from approximately 350 days to 50 weeks and 13 months as we aggregate infection cases (Table 2). Completeness of records also changes for each aggregation level and ultimately affects sample size. The effective length of a time series, *N_E_*, is the maximum number of temporal units in the study period, *T*. Due to missing or incomplete data, *I_D_*, the actual length or sample size, *N (N = N_E_ − I_D_)*, could be shorter than the effective length. Aggregation effectively reduces the percentage of missing data from approximately 1% for the daily time series to no missing data for the weekly and monthly time series (Table 2).

## 4. Assessing Data Irregularities

### 4.1. Calendar Effects for Daily Data

**Figure 1:**
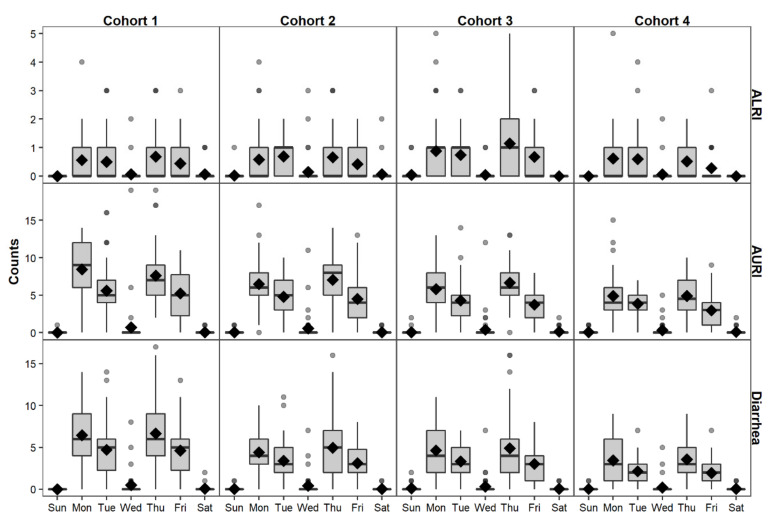
Daily reported cases of ALRI, AURI, and diarrhea per day of the week for each cohort.

Characteristics of a calendar and study design working day definitions must be considered when analyzing time-series data. The definition of working and non-working days can significantly affect data collection procedures. Figure 1 shows almost no reported cases of diarrhea, AURI, or ALRI on Wednesdays, Saturdays, and Sundays. Saturdays and Sundays are considered non-working days in Quito, Ecuador. Wednesday is traditionally a working day, but the team had meetings on Wednesdays to evaluate the fieldwork. Since no data collection occurred on Wednesdays, Saturdays, and Sundays, we grouped them into a non-working day category. We created a day of the week (DoW) variable that included either two categories (working and non-working days) or five categories (Monday, Tuesday, Thursday, Friday, and non-working days). We ran a Poisson regression model (see Section 7.1 for model selection) with a variable for DoW to compare the effects of grouping working days into two rather than five categories. Model results were comparable and explained between approximately 19% to 65% of daily data variability [Equation (2) in Section 7.2] but grouping working days together into a variable with two categories increases the degrees of freedom of the model (Table S1). Thus, DoW was considered for the rest of this tutorial to have two categories (working and non-working days).

### 4.2. Aggregation Irregularities for Weekly and Monthly Data

When aggregating data, some of the weeks and months had missing days. A strategy for dealing with missing or incomplete records is to first calculate the average number of counts per day based on available data and then multiply that by the expected number of days in the week or month. This type of correction for incomplete weeks should not be done without careful consideration of the underlying data structure and the data collection scheme, especially if the missing data are not random. As mentioned in Section 4.1, this study had three non-working days and four working days in a week. Non-working days are expected to have zero counts since data were usually not collected on those days. We cannot ignore this information when handling aggregation of incomplete weeks or we risk overestimating the expected number of cases in a week. Table 3 shows weeks and months that have missing days and the number of missing working and non-working days. Once we determined the number of missing working and non-working days in a time unit, we can determine if that week or month should be considered as complete or incomplete. Only weeks or months that have missing working days are considered as incomplete. For example, Cohort 2 had three weeks with missing days, but only one of those weeks (week 50) had missing working days. Thus, only week 50 was coded as being incomplete. Table 2 and Table 3 show the final number of time units that had aggregation irregularities. The last week of Cohorts 2 and 3 and the last month for all cohorts had missing working days and were considered incomplete.

Once we determine which time units are incomplete, we must check the composition of those weeks or months. Table 4 shows the number of complete and incomplete time units that have zero observed cases of ALRI, AURI, and diarrhea. The incomplete week for Cohort 3, for example, had data for two out of four working days (Table 3), but there were no observed cases for any of the diseases during those working days (Table 4). If all the incomplete weeks or months in a time series have zero observed cases, we cannot run a model to check for differences between complete and incomplete time units. We ran a Poisson regression model (see Section 7.1 for model selection) with an indicator variable for data irregularities only for cohorts that had non-zero observations in at least one incomplete week or month. Models of aggregation irregularities (Table S2) show that as expected, incomplete weeks and months have a lower number of disease counts than complete weeks and months, and those models explained approximately up to 73% of data variability [Equation (2) in Section 7.2].

## 5. Visualizing the Time Series

**Figure 2:**
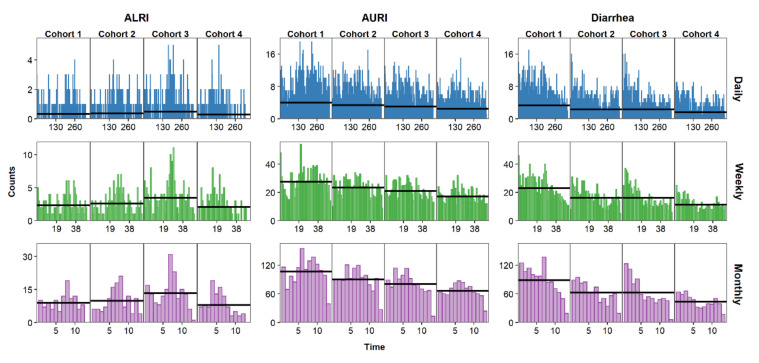
Time series of daily (blue), weekly (green), and monthly (purple) counts of AURI, ALRI, and diarrhea. Black horizontal lines represent the mean for each cohort.

Once the daily, weekly, and monthly time series have been created, it is essential to visualize them to identify characteristics that will inform the statistical analysis, such as temporal trends and seasonal patterns. Needle time series plots represent the relationship between time on the horizontal axis and the outcome on the vertical axis. Figure 2 shows the time series needle plots of daily, weekly, and monthly counts of ALRI, AURI, and diarrhea. The horizontal alignment of graphs in this figure allows for comparison across infections while vertical alignment allows for comparison across aggregation levels. AURI and ALRI show clear seasonal patterns with characteristic peaks (Figure 2). Cases for diarrhea started generally high with each new cohort and decreased over time, likely due to increased immunity with age in children [35]. Data aggregation may affect our perception of trends and seasonality. The seasonal pattern of ALRI cases, for example, is more apparent in the monthly than in the daily time series. By choosing to aggregate data from daily to weekly or monthly counts, the researcher is essentially smoothing over the short-term variability to reveal the underlying temporal patterns of the data.

Calendar plots, radar plots, and box plots stratified by month are also used to visualize time series data [16]. Regardless of the visualization techniques used, interpretation of the time series plots, as outlined above, already provides clues to the directions that a researcher must take during statistical model building. A model that includes trend and seasonality parameters will likely explain more data variability for monthly and weekly aggregated data than for daily data. For more granular data, the researcher will need to include additional modeling parameters, such as a DoW parameter to account for short-term data variability.

## 6. Characterizing the Data Distribution

**Figure 3:**
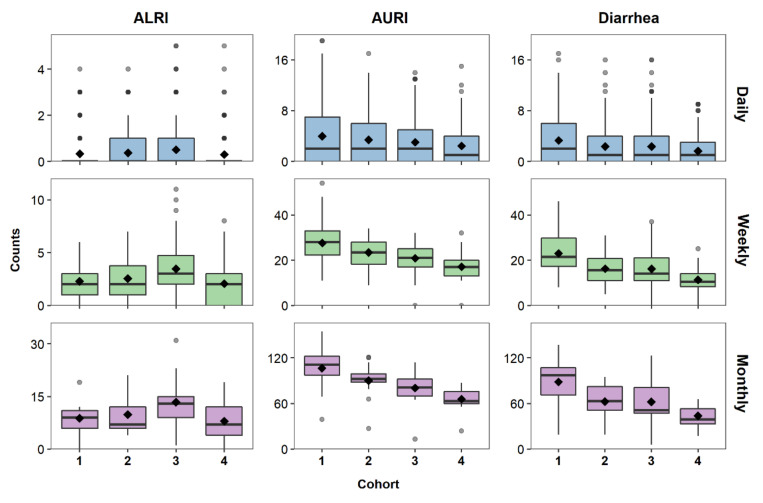
Boxplots of daily (blue), weekly (green), and monthly (purple) counts of ALRI, AURI, and diarrhea for each cohort. Mean counts are shown as black diamonds.

After creating and visualizing the time series, the data can be explored for basic summary and distributional features using box plots. Figure 3 shows box plots of disease counts for each cohort and aggregation level. The median is lower than the mean for daily counts of all diseases, indicating a right-skewed distribution that is often characteristic of discrete health outcomes. ALRI, a less common infection than AURI and diarrhea, has the fewest number of reported cases with Cohorts 1 and 4 having mostly zero cases for the daily time series. This variability between cohorts is smoothed out when aggregating ALRI data to weekly and monthly cases. AURI and diarrhea, on the other hand, show a greater variability between cohorts as the data is aggregated from daily to weekly and monthly cases (Figure 3). These differences between cohorts show that there is a need to further investigate and model temporal distributions of each cohort individually.

Descriptive statistics complement visual representations of data distributions by providing a general sense of central tendency and spread. Measures of central tendency, including the mean and the median, provide estimates of the central location of a distribution. Measures of spread describe the shape and variability of the distribution; and they include the minimum, maximum, standard deviation (sd), variance, coefficient of variation (CV), skewness, and kurtosis. The mean, variance, skewness, and kurtosis, also known as the conventional moments of a distribution, provide information about the shape of that distribution but are very sensitive to outliers. L-moment ratios (L-CV, L-skewness, and L-kurtosis) are analogous to conventional moments but are less sensitive to outlier influence, and are thus particularly valuable for skewed data and small sample sizes [36]. Descriptive statistics, including moments and L-moment ratios for each cohort and aggregation level, are shown in Table 5 for ALRI and in Table S3 for AURI and diarrhea. Descriptive statistics for ALRI are similar for each cohort regardless of aggregation. Although we observed Cohorts 1 and 4 had lower daily reported cases of ALRI (Figure 3), Table 5 shows that those differences are minor. For example, the median for all cohorts was 0 and the mean ranged from 0.330 (sd = 0.685) reported cases of ALRI in Cohort 1 to 0.506 (sd = 0.907) cases for Cohort 3. AURI and diarrhea also show similar yearly statistics for daily counts (Table S3). However, just as observed in the box plots (Figure 3), reported cases of AURI and diarrhea show more variability between cohorts for weekly and monthly aggregation. Aggregation also impacts the proportion of time with no reported cases, especially for rare infections. The proportion of time with no reported cases for ALRI, a less common infection than AURI and diarrhea, decreases from an average of approximately 75% for daily aggregation to 16% and 4% for weekly and monthly aggregation, respectively. AURI and diarrhea show approximately 40% of days with no reported disease cases as compared to 0% of months with no reported cases (Table S3).

Aggregation strongly affects the estimates of standard deviation, variance, CV, skewness, kurtosis, L-CV, L-skewness, and L-kurtosis because their calculations depend on sample size. A higher level of aggregation results in a lower sample size (as seen in Table 2) and less variability observed in the data. The CV, a unitless measure of dispersion, is calculated by dividing the standard deviation by the mean. The higher the CV, the greater the dispersion in the variable. For all infections in our study, the CV decreases by approximately 60% to 80% when aggregating disease counts from daily to weekly or monthly (e.g., CV = 2.074 for daily and 0.509 for Cohort 1 monthly cases of ALRI; Table 5).

**Figure 4:**
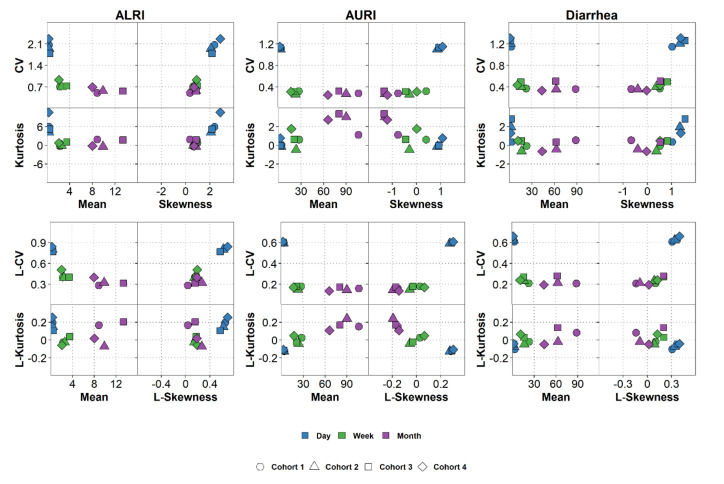
Plots of moments and L-moment ratios of daily, weekly, and monthly counts of AURI, ALRI, and diarrhea.

To explore patterns in the calculated descriptive statistics, we created moment plots and L-moment plots (Figure 4). The moment plots include information on mean, CV, skewness, and kurtosis; and the L-moment plots on mean, L-CV, L-skewness, and L-kurtosis. We can clearly see that CV and L-CV values are the highest for daily data but there is not much of a difference between weekly and monthly data for all infections. Skewness and kurtosis or the analogous L-skewness and L-kurtosis are also measures of data variability, but they are particularly valuable for understanding the behavior of time series data. High values of skewness and kurtosis (or L-skewness and L-kurtosis) are indicative of specific temporal behavior, where diseases exhibit periods of low incidence alternating with periods of high incidence or potential outbreaks. Large kurtosis (or L-kurtosis) values may also indicate the presence of spikes in the time series. Daily data for all infections have the highest values of L-skewness compared to weekly and monthly data regardless of the infection. The L-moment plots thus show that aggregating daily data into weekly counts impact the data distribution (e.g., L-CV and L-skewness decrease) regardless of the rarity of an infection.

## 7. Modeling Time Series

We begin the model building process by selecting the distribution that we will use to model the outcome; we then consider trend, seasonality, and data irregularities; and calculate peak timing and amplitude. Throughout this process, we examine how temporal aggregation may affect model results.

### 7.1. Selecting a Model

A Poisson distribution is commonly used to model discrete counts of health outcomes and has an assumption of mean-variance equality. Data that have spikes and seasonal patterns could violate that assumption and result in over-dispersion relative to a Poisson model. In our data, the mean and variance are closest in value for daily aggregation, with ALRI having the closest values (Table 5 and Table S3). As data are aggregated from daily to monthly, variance becomes higher than the mean. For example, the ratio of variance over the mean for all cases of diarrhea increases from approximately 3.6 to 10.1 as data are aggregated from daily to monthly counts. For weekly and monthly aggregation, the Poisson model could display over-dispersion since the variance is higher than the mean. To adjust for over-dispersion, a researcher may use a quasi-Poisson or a negative binomial distribution, which includes a parameter that accounts for over-dispersion [10,37]. For ease of comparison, we will use in this tutorial a Poisson model for all infections and aggregation levels.

In a Poisson regression adapted to time series data, we regress a health outcome variable against a time-referencing variable:

(1) $$\ln\left(E\left[Y\left(t\right)\right]\right)=f\left(t\right)$$

where *E[Y(t)]* is the expected value of disease counts at time t; and *f(t)* are the time-referencing variables associated with seasonality, trend, and irregular patterns.

### 7.2. Assessing Model Fit

We assessed the overall model fit based on variability explained by the model, the Akaike information criterion (AIC) and the Bayesian information criterion (BIC), and estimates, confidence intervals (CIs), and *p*-values of model parameters. We calculated variability explained by each model from null and residual deviances [Equation (2)]:

(2) $$\mathrm{Variability}\;\mathrm{explained}\;\left(\%\right)=\frac{\mathrm{Null}\;\mathrm{deviance}\;-\;\mathrm{Residual}\;\mathrm{deviance}}{\mathrm{Null}\;\mathrm{deviance}}\times 100$$

As a visual check, we also plotted the predicted model curve over the time series data. Visual inspection allows us to compare model predicted values to trends observed in the data. Although not specifically addressed in this tutorial, we recommend researchers assess the quality of model fit by also inspecting residual plots and testing potential violations to model assumptions [10].

### 7.3. Modeling Seasonality, Trend, and Data Irregularities

Seasonality refers to systematic periodic fluctuations in an outcome of interest. The periodic structure implies that the behavior repeats itself at regular intervals or periods. A harmonic model describes the periodic seasonal curve using a cosine function with symmetric rise and fall over a period of time [6,9]:

(3) $$Y_{t}=\gamma \cos\left(2\pi \omega t-\psi \right)+\varepsilon$$

where $Y_{t}$ is a time series, γ is the amplitude, ω the frequency, ψ the phase angle of the periodic component, and ε is an error term. This harmonic model is easy to interpret but difficult to estimate since it has two unknown parameters, γ and Ψ. Since$\;cos\left(\alpha -\beta \right)=cos\alpha \;cos\beta +sin\alpha \;sin\beta$, we can transform the harmonic model to an equivalent model that is easy to fit by least-squares regression:

(4) $$Y_{t}=\beta_{c}cos\left(2\pi \omega t\right)+\beta_{s}sin\left(2\pi \omega t\right)+\varepsilon$$

where $\beta_{c}=\gamma \cos\left(\psi \right)$ and $\beta_{s}=\gamma \sin\left(\psi \right)$, and all other terms are the same as Equation (3).

In this tutorial, we used Poisson harmonic regression to characterize seasonality of ALRI, AURI, and diarrhea:

(5) $$ln\left(E\left(Y_{t}\right)\right)=\beta_{0}+\beta_{c}cos\left(2\pi \omega t\right)+\beta_{s}sin\left(2\pi \omega t\right)$$

where $Y_{t}$ are ALRI, AURI, or diarrhea cases at time t; $\beta_{0}$ is the intercept; $\beta_{s}$ and $\beta_{c}$ are the coefficients of the harmonic terms; ω is the frequency calculated as 1/M, with M representing the length of the annual cycle. The average number of days in a year is the length of the annual cycle for daily data: $M_{day}=$ 365.25 days. We calculated M for weekly and monthly aggregation by dividing $M_{day}$ by the number of days in a week and month respectively. Thus, $M_{week}=$ 365.25/7 or approximately 52.18 weeks. We defined a lunar month as having 28 days, thus $M_{month}=$ 365.25/28 or approximately 13.04 months. This model applies to a single annual seasonal peak but can be expanded, if needed, to model infections with two seasonal peaks [9].

We further adjusted the model to include a trend component. A trend typically reflects a regular, slowly evolving change in mean response that can be observed over a long time period. The simplest forms are linear or monotonic trends, which represent outcomes that show a relatively steady increase or decrease over time. To model the overall decreasing trend in infections over time we added a linear time term to the model:

(6) $$ln\left(E\left(Y_{t}\right)\right)=\beta_{0}+\beta_{c}cos\left(2\pi \omega t\right)+\beta_{s}sin\left(2\pi \omega t\right)+\beta_{trendL}t$$

where $\beta_{trendL}$ is the coefficient for the linear component of time, and all other terms are the same as Equation (5). If needed, this model can be expanded to include non-linear associations with time, for example by including an additional quadratic term for time to account for acceleration or deceleration in the trend.

Data irregularities, such as DoW for daily data and complete or incomplete aggregation for weekly and monthly data were used for building the time series and must be also considered during modeling. We incorporated data irregularities into the harmonic, trend-adjusted regression model shown in Equation (6):

(7) $$\ln\left(E\left(Y_{t}\right)\right)=\beta_{0}+\beta_{t}t+\beta_{c}cos\left(2\pi \omega t\right)+\beta_{s}sin\left(2\pi \omega t\right)+\beta_{trendL}t+\beta_{IR}IR\left(t\right)$$

where $\beta_{IR}$ reflects the coefficient for the day of the week, *DoW(t)* for daily data or the coefficient for incomplete aggregation for weekly and monthly data, when applicable; and all other terms are the same as for Equation (6). In this same iterative process, a researcher could continue adding additional time-referencing variables relevant to the study (e.g., meteorological parameters).

**Figure 5:**
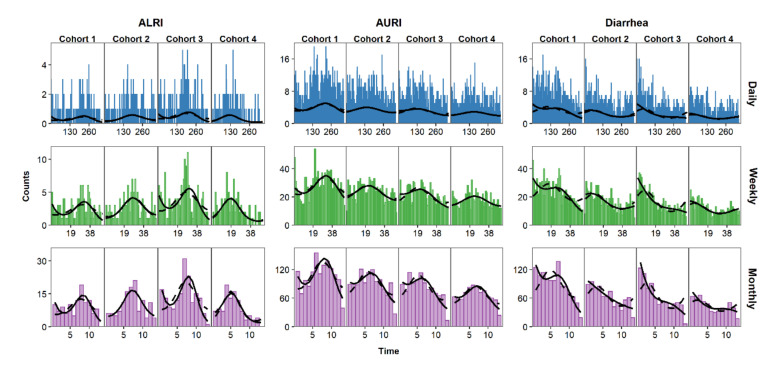
Time series plots showing modeled seasonality as a dashed line and modeled seasonality plus trend as a solid line for daily, weekly, and monthly counts of AURI, ALRI, and diarrhea.

**Figure 6:**
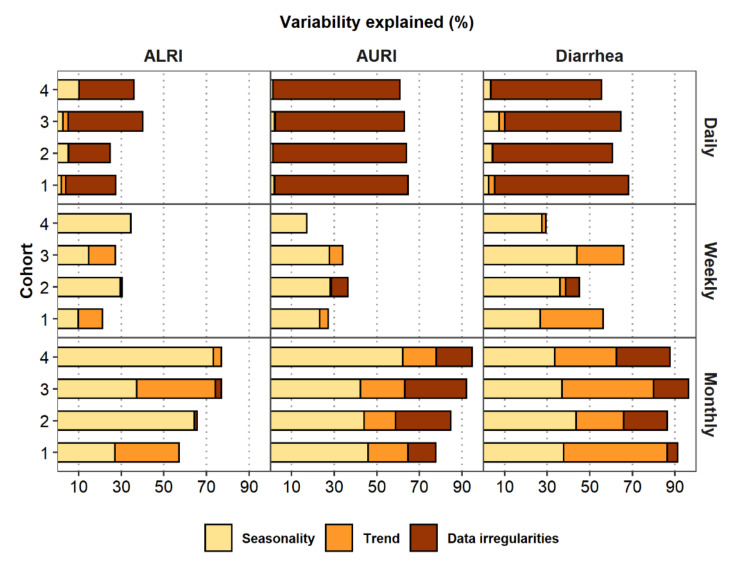
Model variability explained for daily, weekly, and monthly counts of AURI, ALRI, and diarrhea for each cohort.

Results of the seasonal regression model with and without the trend show pronounced seasonal patterns for AURI and ALRI for all cohorts and aggregation levels, with a peak occurring around the middle of each study year (Figure 5). Adding trends to the seasonal models of ALRI and AURI only improved model fit for weekly and monthly aggregation for some cohorts (Figure 6, Table S4). For example, including trend in the monthly ALRI model lowered AIC and BIC, and increased variability explained from approximately 38% to 74% for Cohort 3, but those model fit parameters remained almost the same for Cohort 4. Diarrhea shows a more marked temporal trend than ALRI and AURI, with disease cases that start high for each cohort and generally decrease over time and a less defined seasonal pattern (Figure 5). Adding trend to the seasonal models improved model fit for half of the cohorts for daily and weekly data (Cohort 1 and 3) and of all the cohorts for monthly data (Figure 6, Table S4). Model variability explained increased by as much as 48 percentage points when adding trends to the monthly diarrhea seasonal models.

L-moment plots (Figure 3) showed that data aggregation, not the rarity of a disease, contributed to the main difference in distributions of the data. The greater short-term variability observed in daily data results in lower model variability explained, with daily models remaining at or below 10% for all infections when including only seasonal and trend parameters in the model (Figure 6). The addition of the DoW component to daily models increased variability explained dramatically with some daily models showing higher variability explained than weekly models, and even close to the same variability explained as some monthly models. For AURI, for example, the daily, weekly, and monthly variability explained were approximately 2%, 27%, and 65%, respectively, for Cohort 1 models with seasonal and trend components. The variability explained for the daily model increased to approximately 65% when adding the DoW component to the model (Figure 6). Adding an indicator variable for complete or incomplete aggregation in weekly and monthly data also generally increases the variability explained by the model. The variability explained for monthly AURI data, for example, increased from 65% to 78%. As discussed in Section 4.2, we only accounted for data irregularities in weekly and monthly models that had non-zero counts for at least one irregular week or month.

### 7.4. Characterizing Amplitude and Peak Timing

We calculated the amplitude, γ, and the phase shift, Ψ, of the harmonic regression from estimates of $\beta_{c}$ and $\beta_{s}$ [Equations (8) and (9)] and the CIs as $CI_{\gamma }=1.96*\;\sqrt{Var\left(\gamma \right)}$ and $CI_{PT}=1.96*\;\sqrt{Var\left(\psi \right)}*M/2\pi$, respectively. The variance of the amplitude and peak timing were calculated using the δ-method [Equations (10) and (11)] using the variances ($\sigma_{\beta_{s}}^{2}$ and $\sigma_{\beta_{c}}^{2}$) and the covariance ($\sigma_{\beta_{s}\beta_{c}}$) of the estimates of $\beta_{s}$ and $\beta_{c}$ [1].

(8) $$\gamma =\sqrt{\beta_{s}^{2}+\beta_{c}^{2}}$$

(9) $$\psi =\arctan\left(\frac{\beta_{s}}{\beta_{c}}\right),-\frac{\pi }{2}<\Psi <\frac{\pi }{2}$$

(10) $$Var\left(\gamma \right)≅\frac{{\left(\beta_{s}\sigma_{\beta_{s}}\right)}^{2}+{\left(\beta_{c}\sigma_{\beta_{c}}\right)}^{2}+2\beta_{s}\beta_{c}\sigma_{\beta_{s}\beta_{c}}}{\beta_{c}^{2}+\beta_{s}^{2}}$$

(11) $$Var\left(\psi \right)≅\frac{{\left(\beta_{s}\sigma_{\beta_{s}}\right)}^{2}+{\left(\beta_{c}\sigma_{\beta_{c}}\right)}^{2}-2\beta_{s}\beta_{c}\sigma_{\beta_{s}\beta_{c}}}{{\left(\beta_{c}^{2}+\beta_{s}^{2}\right)}^{2}}$$

**Figure 7:**
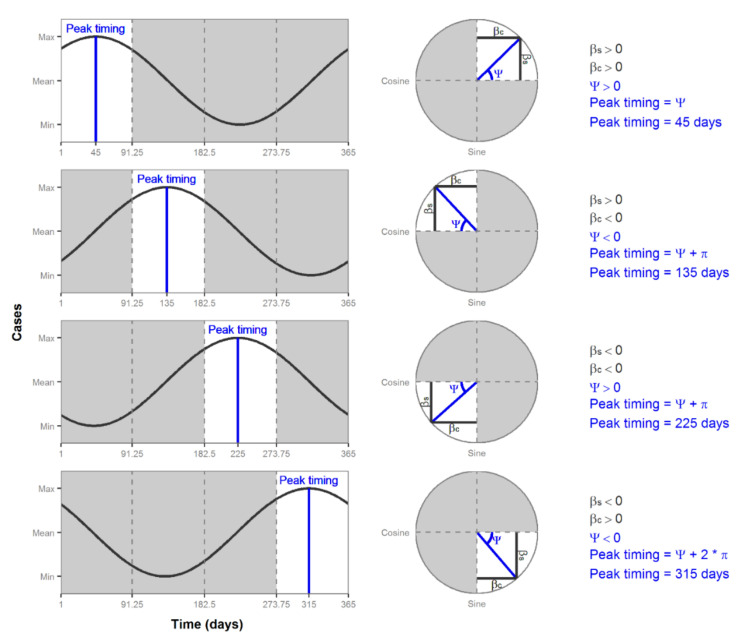
Peak timing plots and formulas for four quadrants.

The estimate of the phase shift, ψ, is in radians and it is only defined in the range −π/2 < Ψ < π/2. To estimate peak timing in time units, we convert the phase shift estimate to a unit between 0 and 2π radians, and then multiply it by M/2π. The circumference of a unit circle is 2π, which corresponds to M, the length of an annual cycle (e.g., 365 days for non-leap years). The formulas used to convert the estimate of the phase shift to peak timing depends on the sign of the $\beta_{s}$ and $\beta_{c}$ estimates. Coefficients $\beta_{s}$ and $\beta_{c}$ are positive or negative based on their quadrant location on a circle. Figure 7 shows the four quadrants of a circle and their corresponding location in a time series plot. In the first quadrant, $\beta_{s}$ and $\beta_{c}$ are both positive, which results in a positive estimate of ψ. Figure 7 shows that estimate as an angle starting from 0. Thus, to calculate the peak timing in the first quadrant, we simply multiply the estimate of ψ by M/2π. In the second and third quadrants, $\beta_{c}$ is negative, which results in the estimate of ψ being an angle starting from π. Since we need an angle starting from 0, we add π to the estimate of ψ and then multiply it by M/2π to calculate peak timing. The fourth quadrant has a negative $\beta_{s}$ and a positive $\beta_{c}$, resulting in a negative estimate of ψ, which is shown in Figure 6 as an angle measure from 2π. Thus, to calculate the peak timing in the fourth quadrant, we add 2π to the estimate of ψ and multiply it by M/2π. Table 6 summarizes the formulas needed to calculate peak timing from an estimate of the phase shift.

**Figure 8:**
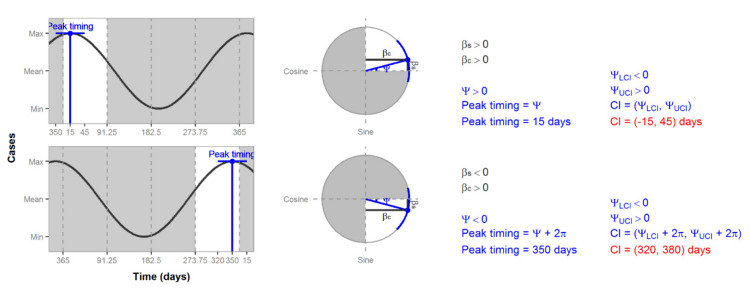
Plots of peak timing CIs. Values that fall outside of the shown year range (1 to 365 days) are highlighted in red.

We calculated the CIs of peak timing using Equation (11). Since peak timing is defined only for one year, we must be aware of times when the CI falls on the edge of that range. Figure 8 shows two estimates of peak timing that are on the edge of the first quadrant and on the edge of the fourth quadrant. If we calculate the CIs, as usual, we would get values that are not possible, e.g., 15 days or 380 days (Figure 8). In this case, we must add or subtract M (365 days in the case of Figure 7 and Figure 8) to correct the CI estimates to have a value between 1 and M.

## 8. Comparing Model Results

**Figure 9:**
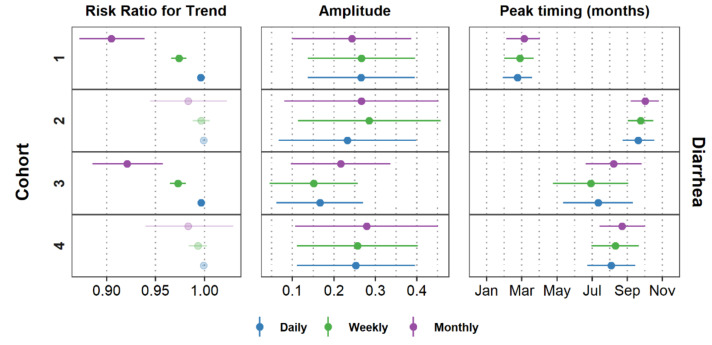
Estimates and confidence intervals for trend, amplitude, and peak timing of diarrhea for all aggregation levels. Faded symbols indicate estimates that had a *p*-value > 0.05. Estimates for ALRI and AURI are shown in Figure A1 in the Appendix A.

**Figure A1:**
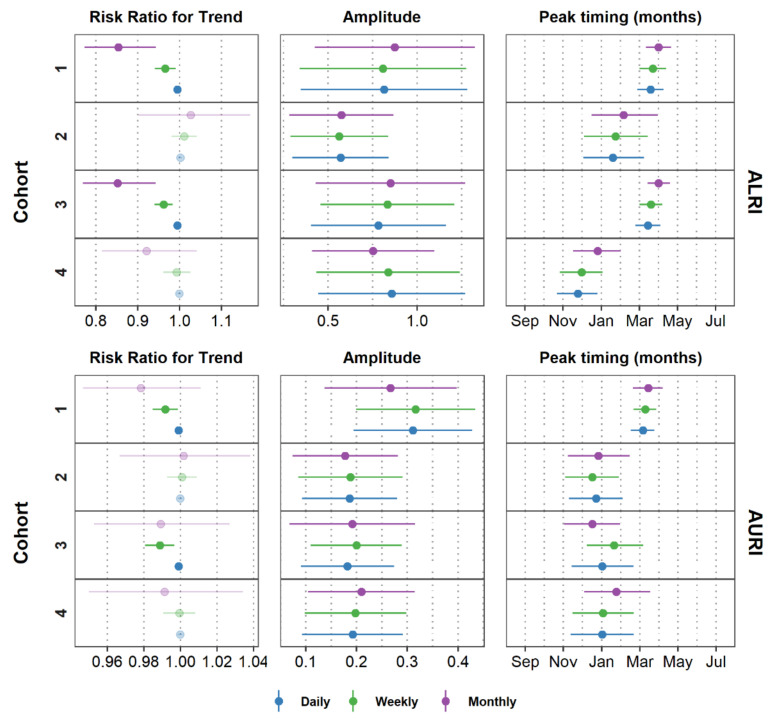
Trend and seasonal harmonics model fit results for daily, weekly, and monthly time series of ALRI and AURI.

Figure 9 and Figure A1 show the peak timing, amplitude, and trend of ALRI, AURI, and diarrhea for all models after adjusting for data irregularities, where applicable. Data aggregation from daily to weekly and monthly counts affects the estimates of trends for all infections. The magnitude of the effect and the CIs become larger as we aggregate data into coarser time units. The effect on trends is expected since aggregation modified the time unit of analysis. For Cohort 1, for example, cases of diarrhea decreased by approximately 0.4% per day (risk ratio = 0.996; 95% CI = 0.995, 0.997; *p*-value < 0.001) and by 9.5% per month (risk ratio = 0.905; 95% CI = 0.872, 0.939; *p*-value < 0.001). A marked declining trend for all aggregation levels is observed in Cohorts 1 and 3 for diarrhea (Figure 9) and ALRI (Figure A1). For AURI, Cohorts 1 and 3 did not show a consistent declining trend for all aggregation levels (Figure A1). AURI cases for Cohort 1, for example, decreased between 0.1% and 1.5% per week (risk ratio = 0.992; 95% CI = 0.985, 0.999; *p*-value = 0.018), but could decrease by up to 5.3% or increase by up to 1.1% per month (risk ratio = 0.941; 95% CI = 0.947, 1.011; *p*-value = 0.189).

**Figure 10:**
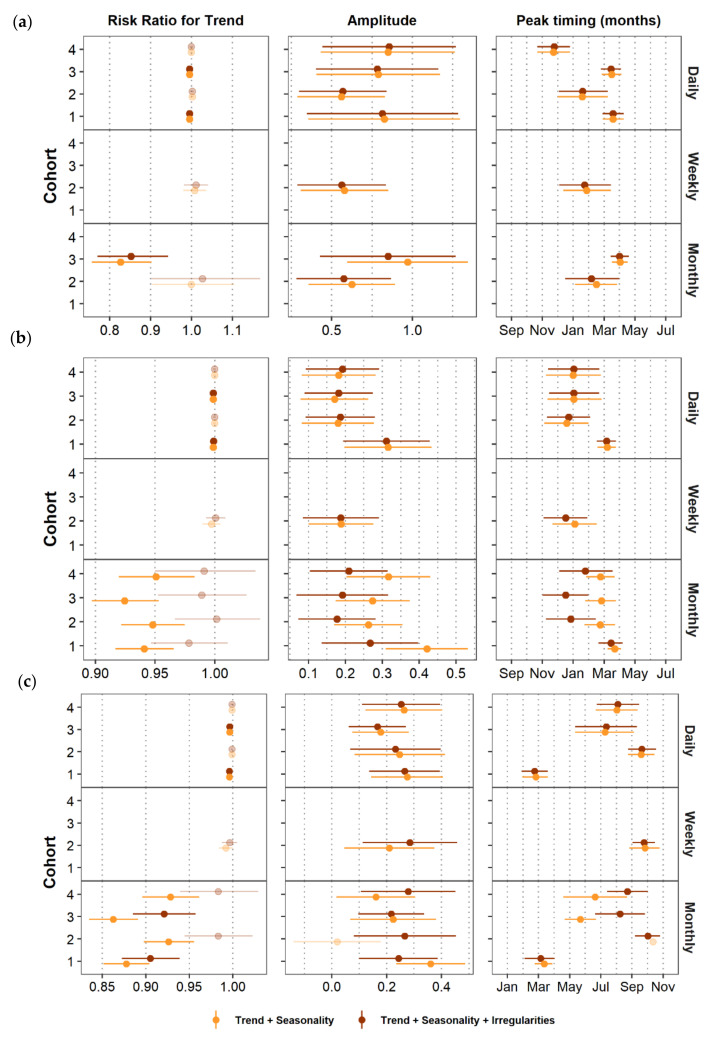
Effect of omitting aggregation irregularities on estimates and confidence intervals for trend, amplitude, and peak timing of (**a**) ALRI, (**b**) AURI, and (**c**) diarrhea for all aggregation levels. Faded symbols indicate estimates that had a *p*-value > 0.05. The confidence interval for peak timing of monthly Cohort 2 data is not shown since it was calculated as 305 days.

Data aggregation showed no impact on the estimates for amplitude and peak timing for any of the diseases, regardless of the strength of the seasonal pattern. As a sensitivity analysis, we compared estimates of trend, peak timing, and amplitude between the full model (trend + seasonality + data irregularities) and a model that did not account for data irregularities. Figure 10 shows that including data irregularities into the model had a minimal impact on the estimates of trend, amplitude, and peak timing for daily and weekly data regardless of the infection. This minimal impact is also observed for monthly ALRI data, the rarest of the three infections in our case study. For the more common infections, AURI and diarrhea, monthly data irregularities affected on average estimates of trend, amplitude, and peak timing. Peak timing estimates for monthly AURI and diarrhea data varied by as much as 2.5 months between the two models. For Cohort 3, for example, the peak timing of monthly diarrhea cases was estimated to occur approximately on May 21st (estimate = 5.677; 95% CI = 4.667, 6.700) for the model with just trend and seasonality and on August 7th (estimate = 8.226; 95% CI 6.633, 9.833) for the model that also accounts for data irregularities. In terms of trend, the magnitude of the estimates for AURI and diarrhea decreased when we controlled the model for monthly data irregularities. Figure 5 and Table 3 showed that the last month in the time series has the lowest number of cases and is considered an incomplete month. If we do not control for data irregularities in the models of the two more common infections, AURI and diarrhea, the trend component will show a steeper slope to try to capture that last incomplete month. For example, Cohort 3 showed monthly diarrheal cases decreased by 13.7% per month (risk ratio = 0.862; 95% CI = 0.835, 0891; *p*-value < 0.001) for the model with trend and seasonality, and by 7.9% for the model that also accounts for data irregularities (risk ratio = 0.921; 95% CI = 0.885, 0.958; *p*-value < 0.001). In summary, aggregating our data into a coarse unit of analysis (e.g., monthly records in this case), could lead to erroneous estimates of trend, peak timing, and amplitude for more common infections. To avoid erroneous model results, we recommend systematically characterizing and controlling for data irregularities that may have been introduced during data aggregation, especially if using a coarser unit of analysis (e.g., month).

## 9. Further Considerations

We focused this tutorial on temporal aggregation, but spatial aggregation is equally important to consider when analyzing time-series data. When locations are aggregated into larger spatial units for statistical analysis, the distribution of the data is likely to change and impact model results just as we observed for temporal aggregation [38,39]. Researchers who aggregate data spatially or temporally should conduct a sensitivity analysis by testing the stability of observed associations based on different aggregation units, just as it was done in this tutorial with daily, weekly, and monthly aggregation units.

In this tutorial, we showed one of the problems with using infection count averages when missing data are not random (Section 4.2) and included a variable for incomplete and complete weeks in our model (Section 7.3). Whether data are missing at random or not determines the types of methods that should be used for data imputation and modeling [40,41]. The method we showed in this tutorial is just one example that could be applied to this data set. We recommend researchers follow the same critical thinking process we showed here by first characterizing their missing data and then selecting an appropriate method for handling their missing data.

The iterative model building process shown in this tutorial serves as a foundation to continue adding additional time-referenced variables relevant to the study. For example, a researcher may want to explore the association of meteorological parameters with infection counts. Adding those additional exposures to the model requires considering the potential of lagged effects, non-linear associations between exposures and disease counts, and exploring effect modifiers [10].

While it could be easier sometimes to illustrate a technique using simulated data with well-predefined properties [42], we intentionally selected a real-life data set that is rich in complexity. With these data, we show the challenges that are common but rarely addressed in epidemiological data analysis. In fact, in our early work, we demonstrated a significant increase in respiratory infections associated with volcanic ashes due to the Pichincha volcano eruption in Quito, Ecuador [43]. We used time series analysis and dealt with data irregularities not addressed by textbooks in epidemiology and biostatistics. The innovations in modern epidemiology will continue to be inspired by irregularities detected by real-world examples of time series data.

## 10. Conclusions

This tutorial presents a critical thinking process for time series analysis with data aggregation. We illustrated the time series analysis process for diseases that have similar seasonal patterns but a different incidence (AURI and ALRI) and diseases that have similar incidence but different seasonal patterns (AURI and diarrhea). We provided a step-by-step procedure for building the time series with careful consideration of data aggregation and temporal references. We observed characteristics of data distributions using multiple visualization and descriptive statistic techniques, including time series plots and L-moment plots. We built time series models accounting for trends, seasonal patterns, and data irregularities for daily, weekly, and monthly data using study characteristics as a temporal reference. Throughout this process, we checked the stability of findings across cohorts. This robust methodical process showed that aggregating data into months impacts model results, particularly for more common infections.

## Figures and Tables

**Table 1:** Daily, weekly, and monthly disease counts based on a calendar and study time references. Diarrheal counts are shown as an example for the first 28 days for Cohort 1. Blue boxes show the study aggregation scheme, while brown boxes show the calendar aggregation scheme.

Date	Day of the Week	Day		Counts of Diarrhea				
				Daily	Weekly		Monthly	
		*Calendar*	*Study*		*Calendar*	*Study*	*Calendar*	*Study*
7/16/2000	Sun.	1			25		…	
7/17/2000	Mon.	2					72	
7/18/2000	Tue.	3						
7/19/2000	Wed.	4						
7/20/2000	Thu.	5	1	16		46		125
7/21/2000	Fri.	6	2	9				
7/22/2000	Sat.	7	3	0				
7/23/2000	Sun.	8	4	0	36			
7/24/2000	Mon.	9	5	14				
7/25/2000	Tue.	10	6	7				
7/26/2000	Wed.	11	7	0				
7/27/2000	Thu.	12	8	9		32		
7/28/2000	Fri.	13	9	6				
7/29/2000	Sat.	14	10	0				
7/30/2000	Sun.	15	11	0	30			
7/31/2000	Mon.	16	12	11				
8/1/2000	Tue.	17	13	6				
8/2/2000	Wed.	18	14	0				
8/3/2000	Thu.	19	15	9		25		
8/4/2000	Fri.	20	16	4				
8/5/2000	Sat.	21	17	0				
8/6/2000	Sun.	22	18	0	19			
8/7/2000	Mon.	23	19	7				
8/8/2000	Tue.	24	20	5				
8/9/2000	Wed.	25	21	0				
8/10/2000	Thu.	26	22	5		22		
8/11/2000	Fri.	27	23	2				
8/12/2000	Sat.	28	24	0				
8/13/2000	Sun.	29	25	0				
8/14/2000	Mon.	30	26	6				
8/15/2000	Tue.	31	27	9				
8/16/2000	Wed.	32	28	0	30		116	
…	….	…	…	…	…	…	…	…

**Table 2:** Sample length for daily, weekly, and monthly aggregation.

Cohort	Effective Length	Actual Length	Missing Data	Aggregation Irregularities
	N_E_	N	I_D_ (%)	n (%)
**Daily Aggregation**				
1	350	348	2 (0.6)	--
2	350	345	5 (1.4)	--
3	350	344	6 (1.7)	--
4	350	348	2 (0.6)	--
**Weekly Aggregation**				
1	50	50	0 (0.0)	0 (0.0)
2	50	50	0 (0.0)	1 (2.0)
3	50	50	0 (0.0)	1 (2.0)
4	50	50	0 (0.0)	0 (0.0)
**Monthly Aggregation**				
1	13	13	0 (0.0)	1 (7.7)
2	13	13	0 (0.0)	1 (7.7)
3	13	13	0 (0.0)	1 (7.7)
4	13	13	0 (0.0)	1 (7.7)

**Table 3:** Weeks and months with aggregation irregularities for the time series.

Cohort	Time	Missing Days (%)			Aggregation Standard	Time Units with Aggregation Irregularities
		Non-Working	Working	Total		
**Weekly Aggregation**						
1	28	1 (33.3)	--	1 (14.3)	Complete	0
	50	1 (33.3)	--	1 (14.3)	Complete	
2	10	2 (66.7)	--	2 (28.6)	Complete	1
	48	1 (33.3)	--	1 (14.3)	Complete	
	50	1 (33.3)	1 (25.0)	2 (28.6)	Incomplete	
3	9	1 (33.3)	--	1 (14.3)	Complete	1
	50	3 (100.0)	2 (50.0)	5 (71.4)	Incomplete	
4	50	2 (66.7)	--	2 (28.6)	Complete	0
**Monthly Aggregation**						
1	7	1 (8.3)	--	1 (3.3)	Complete	1
	13	5 (50.0)	6 (42.9)	11 (36.7)	Incomplete	
2	3	2 (16.7)	--	2 (6.7)	Complete	1
	12	1 (8.3)	--	1 (3.3)	Complete	
	13	5 (50.0)	7 (50.0)	12 (40.0)	Incomplete	
3	3	1 (8.3)	--	1 (3.3)	Complete	1
	13	7 (70.0)	8 (57.1)	15 (50.0)	Incomplete	
4	13	6 (60.0)	6 (42.9)	12 (40.0)	Incomplete	1

**Table 4:** Number of weeks and months that have zero observed cases of ALRI, AURI, and diarrhea. * when all weeks or months had no observed cases.

Cohort	N	Aggregation Irregularities (n)	ALRI		AURI		Diarrhea	
			Time with No Cases (%)		Time with No Cases (%)		Time with No Cases (%)	
			*Incomplete*	*Complete*	*Incomplete*	*Complete*	*Incomplete*	*Complete*
**Weekly aggregation**								
1	50	0	--	6 (12)	--	0 (0)	--	0 (0)
2	50	1	0 (0)	5 (10)	0 (0)	0 (0)	0 (0)	0 (0)
3	50	1	1 (100)*	4 (8)	1 (100)*	0 (0)	1 (100)*	0 (0)
4	50	0	--	15 (30)	--	1 (2)	--	1 (2)
**Monthly aggregation**								
1	13	1	1 (100)*	0 (0)	0 (0)	0 (0)	0 (0)	0 (0)
2	13	1	0 (0)	0 (0)	0 (0)	0 (0)	0 (0)	0 (0)
3	13	1	0 (0)	0 (0)	0 (0)	0 (0)	0 (0)	0 (0)
4	13	1	1 (100)*	0 (0)	0 (0)	0 (0)	0 (0)	0 (0)

**Table 5:** Descriptive statistics for daily, weekly, and monthly time series of ALRI.

Cohort	Total Cases	Time with No Cases (%)	Minimum	Maximum	Median	Mean	Sd	Variance	CV	Skewness	Kurtosis	L-CV	L-Skewness	L-Kurtosis
	**Daily Aggregation**													
1	115	266 (76.4)	0	4	0	0.330	0.685	0.470	2.074	2.384	5.956	0.818	0.652	0.194
2	128	257 (74.5)	0	4	0	0.371	0.720	0.519	1.942	2.078	4.111	0.803	0.622	0.147
3	174	235 (68.3)	0	5	0	0.506	0.907	0.822	1.793	2.178	5.167	0.770	0.573	0.104
4	104	276 (79.3)	0	5	0	0.299	0.677	0.458	2.264	2.864	10.562	0.841	0.694	0.254
	**Weekly Aggregation**													
1	115	6 (12.0)	0	6	2	2.300	1.644	2.704	0.715	0.614	−0.256	0.399	0.148	−0.025
2	128	5 (10.0)	0	7	2	2.560	1.798	3.231	0.702	0.634	−0.092	0.393	0.143	−0.029
3	174	5 (10.0)	0	11	3	3.480	2.549	6.500	0.733	0.967	1.007	0.401	0.174	0.037
4	104	15 (30.0)	0	8	2	2.080	1.936	3.749	0.931	0.902	0.758	0.509	0.191	−0.058
	**Monthly Aggregation**													
1	115	1 (7.7)	0	19	9	8.846	4.506	20.308	0.509	0.345	1.870	0.284	0.036	0.167
2	128	0 (0.0)	4	21	7	9.846	5.580	31.141	0.567	0.852	−0.399	0.323	0.266	−0.074
3	174	0 (0.0)	1	31	13	13.385	7.556	57.090	0.565	0.879	1.689	0.314	0.155	0.203
4	104	1 (7.7)	0	19	7	8.000	5.538	30.667	0.692	0.682	−0.217	0.399	0.185	0.018

**Table 6:** Formulas used to calculate peak timing from an estimate of phase shift ψ , harmonic regression estimates of $\beta_{s}$ and $\beta_{c}$, and the length of the annual cycle M.

Quadrant	Peak Timing
$\beta_{s}>0$ $\beta_{c}>0$	$\Psi \times \frac{M}{2\pi }$
$\beta_{s}>0$ $\beta_{c}<0$	$\left(\Psi +\pi \right)\times \frac{M}{2\pi }$
$\beta_{s}<0$ $\beta_{c}<0$	$\left(\Psi +\pi \right)\times \frac{M}{2\pi }$
$\beta_{s}<0$ $\beta_{c}>0$	$\left(\Psi +2\pi \right)\times \frac{M}{2\pi }$

## Supplementary Materials

The following are available online at https://www.mdpi.com/1660-4601/17/16/5887/s1, Code S1: Tutorial sample code, Data S1: Tutorial sample data, Table S1: Day of the week (DoW) model results for daily time series of ALRI, AURI, and diarrhea, Table S2: Aggregation irregularities model results for monthly time series of AURI, and diarrhea, Table S3: Descriptive statistics for daily, weekly, and monthly time series of AURI and diarrhea.
