# Supplementary material for: Effects of Data Aggregation on Time Series Analysis of Seasonal Infections
Source: Int J Environ Res Public Health. 2020 Aug 13;17(16):5887. doi: 10.3390/ijerph17165887 (PMC7460497; doi:10.3390/ijerph17165887)

Table S11. Day of the week (DoW) model results for daily time series of ALRI, AURI, and diarrhea.

Cohort	DoW	ALRI		AURI		Diarrhea	
		Rate Ratio (95% CI)*	VE (%)	Rate Ratio (95% CI)*	VE (%)	Rate Ratio (95% CI)*	VE (%)
Non-Working (Wed, Sat, Sun) vs Mon, Tue, Thu, and Fri							
1	Mon	2.626 (1.813, 3.609)	24.34	3.493 (3.175, 3.841)	65.47	3.605 (3.226, 4.029)	64.97
	Tue	2.512 (1.687, 3.503)		3.082 (2.757, 3.437)		3.291 (2.906, 3.720)	
	Thu	2.820 (2.026, 3.793)		3.390 (3.071, 3.740)		3.635 (3.257, 4.059)	
	Fri	2.384 (1.544, 3.383)		3.016 (2.689, 3.372)		3.270 (2.884, 3.699)	
2	Mon	2.041 (1.376, 2.779)	20.50	3.326 (2.988, 3.697)	65.06	3.246 (2.854, 3.685)	58.80
	Tue	2.220 (1.573, 2.947)		3.025 (2.680, 3.402)		2.985 (2.584, 3.429)	
	Thu	2.170 (1.520, 2.899)		3.412 (3.076, 3.782)		3.366 (2.976, 3.802)	
	Fri	1.718 (1.008, 2.484)		2.961 (2.616, 3.339)		2.903 (2.500, 3.348)	
3	Mon	3.469 (2.567, 4.670)	36.53	3.344 (2.986, 3.740)	64.18	3.340 (2.942, 3.786)	57.58
	Tue	3.296 (2.383, 4.502)		3.041 (2.676, 3.443)		3.006 (2.598, 3.458)	
	Thu	3.731 (2.841, 4.925)		3.481 (3.125, 3.875)		3.394 (2.997, 3.840)	
	Fri	3.202 (2.280, 4.412)		2.900 (2.531, 3.305)		2.911 (2.500, 3.367)	
4	Mon	3.421 (2.392, 4.848)	27.99	3.328 (2.945, 3.756)	62.15	3.525 (3.032, 4.095)	56.20
	Tue	3.388 (2.356, 4.816)		3.090 (2.700, 3.522)		3.050 (2.543, 3.631)	
	Thu	3.245 (2.201, 4.678)		3.328 (2.945, 3.756)		3.559 (3.068, 4.129)	
	Fri	2.626 (1.506, 4.093)		2.831 (2.433, 3.270)		2.952 (2.440, 3.536)	
Non-Working (Wed, Sat, Sun) vs Mon & Tue and Thu & Fri							
1	Mon & Tue	2.571 (1.807, 3.526)	23.57	3.308 (2.997, 3.652)	62.81	3.460 (3.089, 3.879)	63.02
	Thu & Fri	2.626 (1.865, 3.580)		3.221 (2.908, 3.565)		3.469 (3.098, 3.887)	
2	Mon & Tue	2.134 (1.535, 2.829)	19.65	3.188 (2.859, 3.553)	62.49	3.125 (2.743, 3.556)	56.53
	Thu & Fri	1.970 (1.361, 2.671)		3.212 (2.882, 3.576)		3.161 (2.779, 3.591)	
3	Mon & Tue	3.387 (2.511, 4.573)	35.05	3.204 (2.854, 3.594)	60.64	3.187 (2.798, 3.626)	54.97
	Thu & Fri	3.501 (2.629, 4.686)		3.232 (2.882, 3.622)		3.182 (2.793, 3.621)	
4	Mon & Tue	3.404 (2.413, 4.814)	26.94	3.216 (2.842, 3.637)	59.73	3.315 (2.833, 3.879)	52.09
	Thu & Fri	2.982 (1.970, 4.402)		3.110 (2.735, 3.532)		3.301 (2.818, 3.865)	
Non-Working (Wed, Sat, Sun) vs Working (Mon, Tue, Thu, Fri)							
1	Working	2.598 (1.864, 3.538)	23.55	3.265 (2.959, 3.605)	62.68	3.465 (3.098, 3.879)	63.02
2	Working	2.055 (1.484, 2.732)	19.44	3.200 (2.876, 3.561)	62.48	3.143 (2.769, 3.568)	56.51
3	Working	3.445 (2.587, 4.623)	34.93	3.218 (2.874, 3.603)	60.63	3.184 (2.802, 3.618)	54.97
4	Working	3.215 (2.239, 4.618)	25.68	3.165 (2.797, 3.581)	59.54	3.308 (2.833, 3.866)	52.08

Notes:

* All rate ratios had a p-value < 0.001

CI = Confidence interval

VE = Variability explained

Table S2. Aggregation irregularities model results for monthly time series of AURI, and diarrhea.

Cohort	Aggregation irregularities	ALRI			AURI			Diarrhea		
		Rate Ratio (95% CI)	p-value	VE (%)	Rate Ratio (95% CI)	p-value	VE (%)	Rate Ratio (95% CI)	p-value	VE (%)
Weekly aggregation										
1	Non-standard	--	--	--	--	--	--	--	--	--
2	Non-standard	0.386 (0.022, 1.720)	0.343	1.83	0.380 (0.182, 0.686)	**0.004***	14.36	0.302 (0.108, 0.652)	**0.008***	8.14
3	Non-standard	--	--	--	--	--	--	--	--	--
4	Non-standard	--	--	--	--	--	--	--	--	--
Monthly aggregation										
1	Non-standard	--	--	--	0.348 (0.249, 0.472)	**<0.001***	52.86	0.202 (0.124, 0.308)	**<0.001***	52.30
2	Non-standard	0.387 (0.119, 0.919)	0.062	13.23	0.283 (0.189, 0.406)	**<0.001***	67.89	0.286 (0.175, 0.438)	**<0.001***	41.49
3	Non-standard	0.069 (0.004, 0.308)	**0.008***	38.49	0.151 (0.083, 0.250)	**<0.001***	72.59	0.090 (0.036, 0.182)	**<0.001***	43.15
4	Non-standard	--	--	--	0.345 (0.224, 0.506)	**<0.001***	66.50	0.370 (0.219, 0.579)	**<0.001***	37.51

Notes:
* p-value < 0.05
CI = Confidence interval
VE = Variability explained

Table S3. Descriptive statistics for daily, weekly, and monthly time series of AURI and diarrhea.

Cohort	Total Cases	Time with no cases (%)	Min	Max	Median	Mean	Sd	Variance	CV	Skew	Kurt	L-CV	L-Skew	L-Kurt
**AURI**														
Daily aggregation														
1	1383	142 (40.8)	0	19	2	3.974	4.446	19.766	1.119	0.912	0.031	0.600	0.285	-0.125
2	1171	141 (40.9)	0	17	2	3.394	3.741	13.995	1.102	0.861	-0.132	0.593	0.275	-0.128
3	1046	146 (42.4)	0	14	2	3.041	3.443	11.853	1.132	0.923	-0.029	0.605	0.295	-0.120
4	858	148 (42.5)	0	15	1	2.466	2.835	8.036	1.150	1.071	0.768	0.608	0.303	-0.108
Weekly aggregation														
1	1383	0 (0.0)	11	54	28	27.660	8.891	79.045	0.321	0.398	0.615	0.180	0.029	0.027
2	1171	0 (0.0)	9	34	23.5	23.420	6.091	37.106	0.260	-0.294	-0.474	0.149	-0.055	-0.042
3	1046	1 (2.0)	0	32	21	20.920	6.605	43.626	0.316	-0.444	0.629	0.178	-0.029	-0.024
4	858	1 (2.0)	0	32	17	17.160	5.347	28.586	0.312	0.011	1.763	0.170	0.064	0.047
Monthly aggregation														
1	1383	0 (0.0)	39	155	111	106.385	29.985	899.090	0.282	-0.762	1.120	0.160	-0.154	0.152
2	1171	0 (0.0)	27	121	92	90.077	24.534	601.910	0.272	-1.303	3.011	0.145	-0.194	0.241
3	1046	0 (0.0)	13	114	81	80.462	25.777	664.436	0.320	-1.338	3.367	0.173	-0.171	0.169
4	858	0 (0.0)	24	87	63	66.000	16.442	270.333	0.249	-1.211	2.722	0.136	-0.146	0.108
**Diarrhea**														
Daily aggregation														
1	1149	148 (42.5)	0	17	2	3.302	3.793	14.390	1.149	1.026	0.359	0.610	0.307	-0.104
2	815	147 (42.6)	0	16	1	2.362	2.853	8.139	1.208	1.343	1.941	0.625	0.340	-0.075
3	810	147 (42.7)	0	16	1	2.355	2.975	8.853	1.264	1.560	2.817	0.639	0.372	-0.044
4	569	167 (48.0)	0	9	1	1.635	2.138	4.572	1.308	1.379	1.309	0.661	0.397	-0.043
Weekly aggregation														
1	1149	0 (0.0)	8	46	21.5	22.980	8.508	72.387	0.370	0.514	-0.079	0.210	0.102	-0.020
2	815	0 (0.0)	5	31	15.5	16.300	6.643	44.133	0.408	0.371	-0.617	0.234	0.086	-0.049
3	810	1 (2.0)	0	37	14	16.200	7.972	63.551	0.492	0.827	0.471	0.270	0.202	0.029
4	569	1 (2.0)	0	25	10.5	11.380	4.940	24.404	0.434	0.529	0.488	0.240	0.125	0.065
Monthly aggregation														
1	1149	0 (0.0)	19	137	97	88.385	31.957	1021.256	0.362	-0.671	0.543	0.209	-0.141	0.084
2	815	0 (0.0)	19	95	63	62.692	22.511	506.731	0.359	-0.398	-0.438	0.212	-0.094	-0.021
3	810	0 (0.0)	6	123	51	62.308	31.611	999.231	0.507	0.542	0.344	0.281	0.201	0.140
4	569	0 (0.0)	17	66	39	43.769	14.521	210.859	0.332	-0.033	-0.640	0.195	0.018	-0.049

Table S4. Trend and seasonal harmonics model results for daily, weekly, and monthly time series of ALRI, AURI, and diarrhea.

**Cohort**	**Coefficients (95% CI)**			**p-value**			**VE (%)**	**AICc**	**BIC**
	**Sine**	**Cosine**	**Time**	Sine	Cosine	Time			
				**ALRI**					
Daily aggregation									
1	-0.168 (-0.427, 0.088)	-0.301 (-0.577, -0.032)		0.199	**0.030***		1.8	538	549
2	-0.112 (-0.361, 0.136)	-0.567 (-0.843, -0.302)		0.377	**<0.001***		5.0	560	571
3	-0.041 (-0.250, 0.168)	-0.385 (-0.613, -0.162)		0.703	**<0.001***		2.6	694	705
4	0.769 (0.471, 1.084)	-0.461 (-0.769, -0.164)		**<0.001***	**0.003***		10.1	482	494
1	-0.717 (-1.194, -0.250)	-0.407 (-0.707, -0.120)	-0.005 (-0.009, -0.001)	**0.003***	**0.006***	**0.007***	4.0	532	548
2	0.038 (-0.430, 0.511)	-0.558 (-0.835, -0.291)	0.001 (-0.002, 0.006)	0.872	**<0.001***	0.461	5.2	561	576
3	-0.588 (-0.983, -0.197)	-0.524 (-0.782, -0.278)	-0.005 (-0.009, -0.002)	**0.003***	**<0.001***	**0.001***	5.0	685	701
4	0.694 (0.167, 1.236)	-0.487 (-0.846, -0.156)	-0.001 (-0.006, 0.004)	**0.011***	**0.005***	0.739	10.1	484	499
Weekly aggregation									
1	-0.183 (-0.442, 0.074)	-0.290 (-0.564, -0.021)		0.165	**0.036***		9.9	185	190
2	-0.153 (-0.403, 0.095)	-0.577 (-0.852, -0.313)		0.227	**<0.001***		29.5	180	186
3	-0.073 (-0.284, 0.136)	-0.416 (-0.643, -0.195)		0.493	**<0.001***		14.7	227	233
4	0.722 (0.428, 1.034)	-0.528 (-0.838, -0.229)		**<0.001***	**<0.001***		34.4	174	179
1	-0.722 (-1.202, -0.252)	-0.360 (-0.650, -0.080)	-0.035 (-0.061, -0.010)	**0.003***	**0.013***	**0.008***	21.1	180	187
2	-0.044 (-0.510, 0.424)	-0.578 (-0.853, -0.313)	0.008 (-0.020, 0.035)	0.854	**<0.001***	0.587	30.0	182	189
3	-0.653 (-1.049, -0.264)	-0.516 (-0.763, -0.279)	-0.039 (-0.062, -0.017)	**0.001***	**<0.001***	**<0.001***	27.2	218	224
4	0.625 (0.095, 1.167)	-0.555 (-0.902, -0.234)	-0.007 (-0.041, 0.025)	**0.022***	**0.001***	0.667	34.6	176	183
Monthly aggregation									
1	-0.234 (-0.500, 0.027)	-0.331 (-0.599, -0.068)		0.080	**0.015***		27.0	82	81
2	-0.235 (-0.490, 0.016)	-0.579 (-0.847, -0.322)		0.068	**<0.001***		64.3	74	73
3	-0.167 (-0.382, 0.046)	-0.460 (-0.682, -0.243)		0.125	**<0.001***		37.2	97	96
4	0.586 (0.294, 0.890)	-0.682 (-0.992, -0.386)		**<0.001***	**<0.001***		73.2	68	67
1	-0.835 (-1.314, -0.376)	-0.258 (-0.534, 0.013)	-0.158 (-0.261, -0.060)	**<0.001***	0.064	**0.002***	57.1	76	73
2	-0.237 (-0.674, 0.191)	-0.579 (-0.863, -0.307)	-0.001 (-0.101, 0.101)	0.281	**<0.001***	0.990	64.3	78	75
3	-0.877 (-1.281, -0.489)	-0.413 (-0.644, -0.188)	-0.191 (-0.283, -0.105)	**<0.001***	**<0.001***	**<0.001***	74.1	82	79
4	0.292 (-0.231, 0.811)	-0.693 (-1.014, -0.391)	-0.083 (-0.212, 0.035)	0.269	**<0.001***	0.186	76.9	71	68
				**AURI**					
Daily aggregation									
1	-0.104 (-0.178, -0.031)	-0.195 (-0.273, -0.118)		**0.005***	**<0.001***		1.7	2634	2645
2	0.121 (0.041, 0.201)	-0.144 (-0.228, -0.061)		**0.003***	**<0.001***		1.2	2290	2302
3	0.204 (0.119, 0.289)	-0.123 (-0.212, -0.034)		**<0.001***	**0.007***		2.0	2129	2141
4	0.071 (-0.022, 0.164)	-0.171 (-0.269, -0.073)		0.138	**<0.001***		1.1	1856	1868

1	-0.234 (-0.366, -0.102)	-0.211 (-0.290, -0.133)	-0.001 (-0.002, 0.000)	**<0.001***	**<0.001***	**0.020***	2.0	2630	2646
2	0.103 (-0.040, 0.246)	-0.147 (-0.234, -0.061)	0.000 (-0.001, 0.001)	0.159	**<0.001***	0.768	1.2	2292	2308
3	0.068 (-0.085, 0.221)	-0.156 (-0.252, -0.062)	-0.001 (-0.002, 0.000)	0.384	**0.001***	**0.037***	2.3	2127	2142
4	0.052 (-0.115, 0.220)	-0.174 (-0.275, -0.074)	0.000 (-0.001, 0.001)	0.543	**<0.001***	0.794	1.1	1858	1873
Weekly aggregation									
1	-0.115 (-0.189, -0.041)	-0.190 (-0.267, -0.113)		**0.002***	**<0.001***		23.2	371	376
2	0.105 (0.026, 0.185)	-0.173 (-0.257, -0.090)		**0.010***	**<0.001***		28.0	314	319
3	0.194 (0.109, 0.280)	-0.171 (-0.260, -0.082)		**<0.001***	**<0.001***		27.7	334	339
4	0.061 (-0.032, 0.154)	-0.190 (-0.289, -0.093)		0.200	**<0.001***		17.0	315	320
1	-0.247 (-0.379, -0.115)	-0.198 (-0.276, -0.121)	-0.008 (-0.015, -0.001)	**<0.001***	**<0.001***	**0.018***	27.1	368	374
2	0.062 (-0.081, 0.205)	-0.178 (-0.263, -0.093)	-0.003 (-0.010, 0.005)	0.394	**<0.001***	0.475	28.7	316	322
3	0.016 (-0.135, 0.168)	-0.199 (-0.291, -0.107)	-0.011 (-0.020, -0.003)	0.831	**<0.001***	**0.006***	34.0	329	335
4	0.050 (-0.117, 0.217)	-0.191 (-0.290, -0.093)	-0.001 (-0.010, 0.008)	0.558	**<0.001***	0.876	17.1	317	324
Monthly aggregation									
1	-0.138 (-0.213, -0.063)	-0.240 (-0.316, -0.165)		**<0.001***	**<0.001***		45.9	154	153
2	0.079 (-0.003, 0.160)	-0.258 (-0.340, -0.176)		0.058	**<0.001***		44.0	144	143
3	0.177 (0.091, 0.264)	-0.271 (-0.358, -0.184)		**<0.001***	**<0.001***		42.3	162	161
4	0.010 (-0.085, 0.105)	-0.289 (-0.386, -0.193)		0.839	**<0.001***		62.2	108	107
1	-0.373 (-0.500, -0.248)	-0.194 (-0.272, -0.117)	-0.061 (-0.087, -0.035)	**<0.001***	**<0.001***	**<0.001***	64.7	137	134
2	-0.130 (-0.267, 0.006)	-0.228 (-0.312, -0.144)	-0.054 (-0.082, -0.026)	0.062	**<0.001***	**<0.001***	58.8	134	131
3	-0.129 (-0.276, 0.017)	-0.241 (-0.330, -0.153)	-0.078 (-0.109, -0.048)	0.084	**<0.001***	**<0.001***	63.1	140	137
4	-0.184 (-0.344, -0.025)	-0.257 (-0.356, -0.159)	-0.050 (-0.084, -0.017)	**0.024***	**<0.001***	**0.003***	77.9	103	101
**Diarrhea**									
Daily aggregation									
1	0.245 (0.164, 0.326)	-0.102 (-0.186, -0.017)		**<0.001***	**0.018***		2.5	2304	2315
2	0.311 (0.215, 0.409)	0.167 (0.067, 0.267)		**<0.001***	**0.001***		4.3	1787	1798
3	0.357 (0.260, 0.455)	0.319 (0.217, 0.421)		**<0.001***	**<0.001***		7.4	1780	1791
4	0.186 (0.071, 0.300)	0.285 (0.165, 0.406)		**0.001***	**<0.001***		3.4	1452	1463
1	-0.166 (-0.309, -0.023)	-0.219 (-0.314, -0.126)	-0.004 (-0.005, -0.003)	**0.023***	**<0.001***	**<0.001***	5.3	2259	2274
2	0.205 (0.037, 0.372)	0.139 (0.032, 0.246)	-0.001 (-0.002, 0.000)	**0.017***	**0.011***	0.127	4.5	1787	1802
3	-0.057 (-0.225, 0.112)	0.169 (0.052, 0.284)	-0.004 (-0.005, -0.002)	0.511	**0.004***	**<0.001***	10.1	1747	1763
4	0.060 (-0.138, 0.258)	0.256 (0.130, 0.383)	-0.001 (-0.002, 0.000)	0.552	**<0.001***	0.130	3.6	1452	1467
Weekly aggregation									
1	0.241 (0.160, 0.323)	-0.117 (-0.201, -0.032)		**<0.001***	**0.007***		26.8	364	370
2	0.318 (0.222, 0.416)	0.123 (0.023, 0.223)		**<0.001***	**0.016***		36.0	320	325
3	0.382 (0.284, 0.481)	0.255 (0.154, 0.357)		**<0.001***	**<0.001***		44.0	338	343
4	0.210 (0.096, 0.325)	0.256 (0.136, 0.377)		**<0.001***	**<0.001***		27.6	295	300
1	-0.167 (-0.312, -0.023)	-0.207 (-0.299, -0.116)	-0.027 (-0.034, -0.019)	**0.023***	**<0.001***	**<0.001***	56.3	322	328

2	0.186 (0.018, 0.354)	0.098 (-0.006, 0.202)	-0.008 (-0.016, 0.000)	**0.030***	0.064	0.059	38.6	319	326
3	-0.072 (-0.240, 0.096)	0.133 (0.022, 0.243)	-0.028 (-0.036, -0.019)	0.403	**0.018***	**<0.001***	65.9	298	305
4	0.095 (-0.103, 0.294)	0.238 (0.116, 0.362)	-0.007 (-0.016, 0.003)	0.346	**<0.001***	0.165	29.3	295	302
Monthly aggregation									
1	0.228 (0.145, 0.311)	-0.237 (-0.321, -0.154)		**<0.001***	**<0.001***		37.6	191	190
2	0.341 (0.242, 0.440)	-0.019 (-0.117, 0.079)		**<0.001***	0.704		43.5	146	145
3	0.429 (0.329, 0.530)	0.097 (-0.002, 0.196)		**<0.001***	0.055		36.8	214	213
4	0.246 (0.129, 0.364)	0.108 (-0.009, 0.225)		**<0.001***	0.071		33.6	121	120
1	-0.287 (-0.430, -0.144)	-0.219 (-0.304, -0.134)	-0.131 (-0.161, -0.101)	**<0.001***	**<0.001***	**<0.001***	86.3	116	113
2	0.019 (-0.144, 0.182)	0.004 (-0.095, 0.103)	-0.077 (-0.108, -0.046)	0.815	0.936	**<0.001***	65.9	126	124
3	-0.204 (-0.372, -0.036)	0.092 (-0.011, 0.195)	-0.148 (-0.181, -0.116)	**0.017***	0.078	**<0.001***	80.0	130	127
4	-0.075 (-0.267, 0.116)	0.142 (0.023, 0.261)	-0.075 (-0.110, -0.039)	0.443	**0.019***	**<0.001***	62.5	108	105

Notes:

* p-value < 0.05

CI = Confidence interval

VE = Variability explained

Figure S1. Trend and seasonal harmonics model fit results for daily, weekly, and monthly time series of ALRI, AURI, and diarrhea.

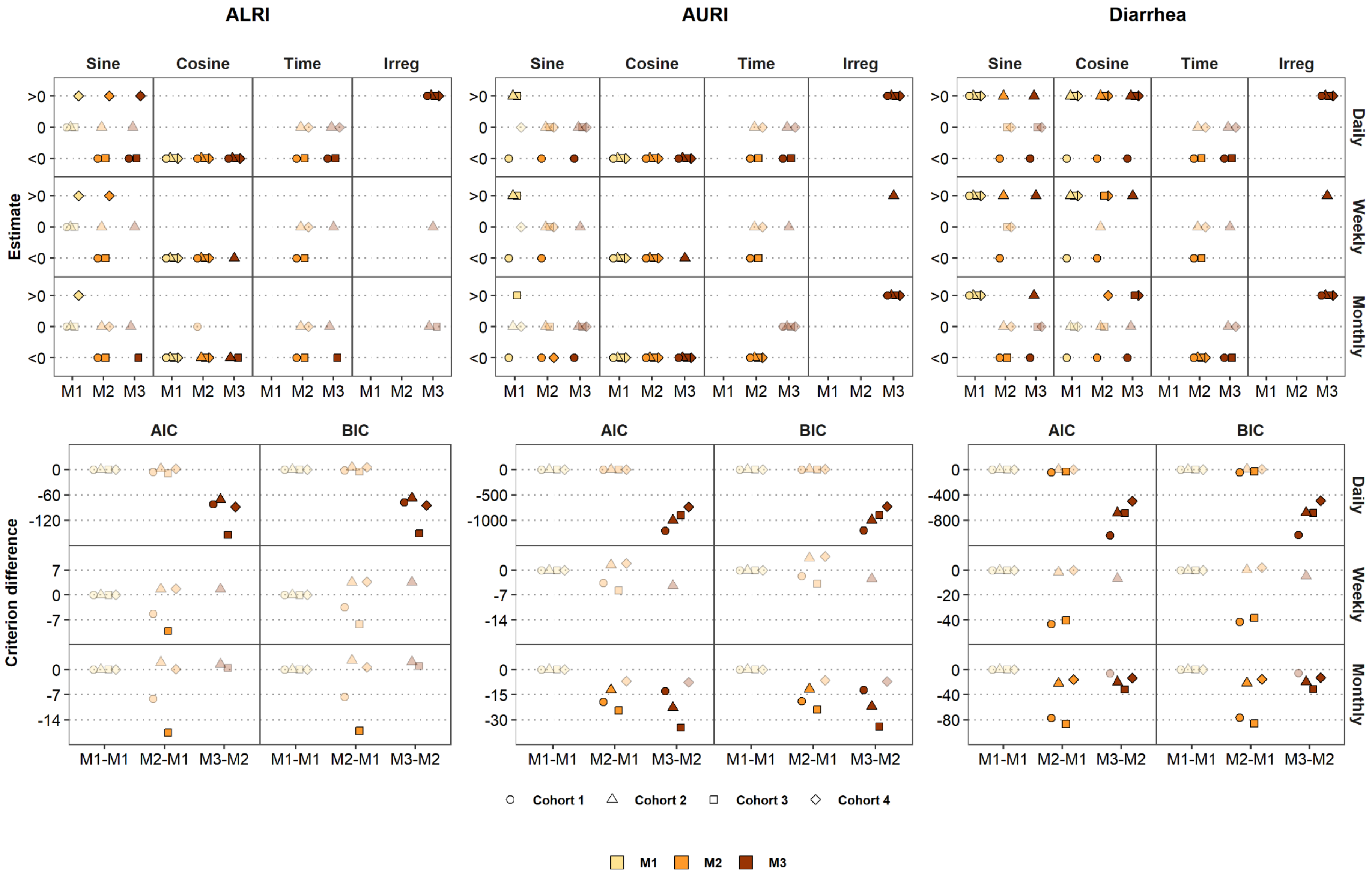

Supplement: Supplementary file 1 [file ijerph-17-05887-s001.pdf]
